# Surgical management and the prognosis of iatrogenic facial nerve injury in middle ear surgery: a 20-year experience

**DOI:** 10.1186/s13005-023-00377-y

**Published:** 2023-07-25

**Authors:** Jianbin Sun, Ruoya Wang, Xingrui Chen, Jianze Wang, Da Liu, Na Sai, Yuhua Zhu, Jun Liu, Weidong Shen, Pu Dai, Shiming Yang, Dongyi Han, Weiju Han

**Affiliations:** 1 Medical School of Chinese PLA, Beijing, 100853 China; 2 Department of Otolaryngology Head and Neck Surgery, the Six Medical Center, Chinese PLA General Hospital, Beijing, 100853 China; 3 National Clinical Research Center for Otolaryngologic Diseases, Beijing, China; 4 Department of Otolaryngology, the First Affiliated Hospital of Jinzhou Medical University, Jinzhou, 121012 China; 5 Department of Medical Oncology, Guangdong SanJiu Brain Hospital, Guangzhou, 510510 China; 6 PLA Air Demonstration Team, Tianjin, 301700 China

**Keywords:** Facial nerve paralysis, Middle ear surgery, Iatrogenic injury, Facial nerve repair

## Abstract

**Background:**

Iatrogenic facial nerve injury is one of the severest complications of middle ear surgery, this study aims to evaluate surgical management and prognosis in the era of improved surgical instruments.

**Methods:**

Patients suffered from facial nerve paralysis after middle ear surgery between January 2000 and December 2019 were retrospectively collected. Demographic characters, primary disease and surgery, details of revision surgery were analyzed.

**Results:**

Forty-five patients were collected, of whom 8 were injured at our center and 37 were transferred.

For 8 patients injured at our center, seven (87.5%) ranked House-Brackmann (H-B) grade V and one (12.5%) ranked H-B VI before revision surgery; postoperatively, two (25.0%) patients recovered to H-B grade I, four (50.0%) recovered to H-B II, and the other two (25.0%) recovered to H-B III. For 37 patients transferred, thirteen (35.1%) ranked H-B grade V and 24 (64.9%) ranked H-B VI preoperatively, final postoperative grade ranked from H-B grade I to grade V, with H-B I 6 (16.2%) cases, H-B II 6 (16.2%) cases, H-B III 18 (48.6%) cases, H-B IV 5 (13.5%) cases and H-B V 2 (5.4%) cases. The most vulnerable site was tympanic segment (5, 62.5% and 27, 73.0% respectively). Twenty-one (46.7%) patients suffered from mild injury and 24 (53.3%) suffered from partial or complete nerve transection. For surgical management, twenty-one (46.7%) patients received decompression, nineteen (42.2%) received graft and 5 (11.1%) received anastomosis. Those decompressed within 2 months after paralysis had higher possibility of H-B grade I or II recovery (*P* = 0.026), those received graft within 6 months were more likely to get H-B grade III recovery (*P* = 0.041), and for patients underwent anastomosis within 6 months, all recovered to H-B grade III.

**Conclusions:**

Tympanic segment is the vulnerable site. If facial nerve paralysis happens, high-resolution computed tomography could help identify the injured site. Timely treatment is important, decompression within 2 months after paralysis, graft and anastomosis within 6 months lead to better recovery.

**Supplementary Information:**

The online version contains supplementary material available at 10.1186/s13005-023-00377-y.

## Introduction

Iatrogenic facial nerve paralysis typically refers to the paralysis that caused by accidental facial nerve injury in surgery. Although iatrogenic facial nerve paralysis is not life-threatening, it has a great impact on patients’ quality of life [1], and sometimes even leads to depression [2]. Therefore, it is taken as one of the severest complications of middle ear surgery. With the advent of advanced surgical instruments, such as facial nerve monitor and high-definition microscope, the incidence of iatrogenic facial nerve paralysis has decreased [3]; however, it is still difficult to completely avoid it, because many factors contribute to its occurrence. Therefore, we retrospectively analyze patients with iatrogenic facial nerve paralysis in the era of improved surgical instruments, in hope of providing clinical experience for otologists to avoid it in middle ear surgery, and properly manage it in case it happens.

## Methods

### Patients

Patients who were hospitalized in our center (a tertiary referral center) from January 2000 to December 2019 due to facial nerve paralysis after middle ear surgery were retrospectively collected. The inclusion criteria were as follows: (1) suffered from facial nerve paralysis immediately after middle ear surgery; (2) received revision surgery for facial nerve paralysis; (3) followed up for no less than 2 years. Those who received surgery for facial nerve tumor or malignant tumor in middle ear in which facial nerve had to be sacrificed were excluded.

### Preoperative investigation and evaluation

Before revision surgery, patient demographic characters (gender, age and side), primary disease and surgery were investigated. Facial nerve function was assessed by at least two otologists using House-Brackmann (H-B) Grade Scale. The site of the facial nerve injury was pinpointed by preoperative high-resolution computed tomography (HRCT) and inoperative exploration. Electromyogram (EMG) and audiometry were routinely conducted.

### Revision surgery procedure for iatrogenic facial nerve paralysis

The revision surgery for iatrogenic facial nerve paralysis was performed by experienced otologists of facial nerve disease team in our center. Retroauricular approach was usually adopted. Residual lesions such as cholesteatoma or granulation, as well as fibrous tissues adjacent to facial nerve, were first cleared. The injured portion of facial nerve was then exposed and repaired. The inner ear injury and ossicular chain were also explored. Concurrent ossicular chain reconstruction was conducted only when there was intact inner ear, normal middle ear mucosa and eustachian tube function. Then if posterior wall of external auditory canal was intact, facial nerve was covered only with temporal fascia and gelatin sponge. If posterior wall was down, skin flap of external canal was transferred to cover the facial nerve. If patient suffered from sensorineural hearing loss and received canal wall down mastoidectomy, then mastoid cavity, middle ear and eustachian tube were obliterated using abdominal fat, with blind-sac closure of the external auditory canal. All the patients received antibiotics and corticosteroid after revision surgery.

### Management of the injured facial nerve

Surgical management for different degrees of facial nerve injury varied.

Decompression was performed if facial nerve trunk was continuous. If fallopian canal was defective and facial nerve was edematous, drilled the canal until more than 5 mm of normal neural trunk appeared. If nerve sheath was injured but axons were intact, just reset the sheath. If facial nerve was superficially transected (less than 1/3 of the circumference), axons were realigned.

Anastomosis was performed for partially (more than 1/3 of the circumference) or completely transected facial nerve. If facial nerve was partially transected, after realigning axons, nerve was anastomosed with monofilament 9/0 thread through the epineurium. If facial nerve was completely transected, the traumatic neuroma on the nerve ending, if existed, was first cleared, then direct anastomosis or rerouting and end-to-end tension-free anastomosis was performed.

Great auricular nerve graft was conducted when end-to-end tension-free anastomosis was infeasible due to big gap between two nerve endings.

### Analysis of clinical characteristics

Three aspects of clinical characteristics were analyzed: (1) patient demographic characters (gender, age and side); (2) primary disease and primary surgery type (only for patients injured at our center); (3) revision surgery details (preoperative H-B grade, preoperative hearing, duration before revision surgery, site of facial nerve injury, degree of facial nerve injury, malformation of facial nerve, presence of traumatic neuroma, residual lesions and inner ear injury, facial nerve management and final postoperative H-B grade).

### Statistical analysis

Quantitative variables were presented as mean and standard deviation, qualitative variables were expressed as a percentage and Fisher’s test was used to determine statistical differences. Two-sided *P* values < 0.05 were considered statistically significant. Analysis was performed with *SPSS 26.0* (SPSS, Chicago, IL, USA), graphs were depicted and processed in *GraphPad Prism 8.0* (GraphPad Software, Los Angeles, CA, USA) and *Adobe Illustrator CC 2020* (Adobe, San Jose, CA, USA).

## Results

### Patient demographic characters, primary disease and surgery type

Forty-five patients were collected, of whom 8 were injured at our center and 37 were transferred.

For 8 patients injured at our center, two (25.0%) were male and 6 (75.0%) were female. Median age was 35.3 ± 16.5 (range 5–53) years old. Six (75.0%) patients paralyzed on the right and 2 (25.0%) on the left. Primary diseases included cholesteatoma of middle ear (4, 50.0%), chronic otitis media (3, 37.5%) and congenital atresia of external auditory canal (1, 12.5%). Besides, four (50.0%)patients received tympanoplasty (one combined with canalplasty), three (37.5%) received canal wall down mastoidectomy (including Bondy’s modified radical mastoidectomy), and one (12.5%) received canal wall up mastoidectomy. From January 2000 to December 2019, thirteen patients suffered from iatrogenic facial nerve paralysis after primary middle ear surgery in our center (5 patients did not meet the inclusion criteria), of whom 7 happened between January 2000 and December 2009, and 6 between January 2010 and December 2019. During the first decade of this century, approximate 5491 middle ear surgeries (exclusive of surgeries for facial nerve tumor or malignant tumor in middle ear in which facial nerve had to be sacrificed) were performed at our center, so the incidence in the first decade of the twenty-first century was about 0.13% (7/5491). The second decade witnessed a dramatic decline on the incidence, which was about 0.04% (6/14552) (Table 1 and Supplementary Table 1).

**Table 1:** Analysis of 8 patients injured at our center

Clinical characteristics	
Gender	
male	2 (25.0%)
female	6 (75.0%)
Age (years)	35.3 ± 16.5 (5–53)
Side	
right	6 (75.0%)
left	2 (25.0%)
Primary disease	
cholesteatoma of middle ear	4 (50.0%)
chronic suppurative otitis media	3 (37.5%)
congenital atresia of external auditory canal	1 (12.5%)
Type of primary surgery	
wall down mastoidectomy	3 (37.5%)
wall up mastoidectomy	1 (12.5%)
tympanoplasty (one case combined with canalplasty)	4 (50.0%)
Incidence^a^	
2000–2009	0.13% (7/5491)
2010–2019	0.04% (6/14552)
Preoperative grade (H-B)	
V	7 (87.5%)
VI	1 (12.5%)
Preoperative hearing	
conductive hearing loss	3 (37.5%)
mixed hearing loss	5 (62.5%)
Duration before revision surgery	
< 2 months	6 (75.0%)
2–6 months	2 (25.0%)
Site of facial nerve injury^b^	
tympanic segment	5 (62.5%)
geniculate ganglion	2 (25.0%)
fallopian canal intact	2 (25.0%)
Degree of facial nerve injury	
canal intact and mild edema	2 (25.0%)
edema	4 (50.0%)
sheath defective and edema	1 (12.5%)
completely transected	1 (12.5%)
Malformation of facial nerve	
fallopian canal dehiscent	5 (62.5%)
anomalous course	1 (12.5%)
Inner ear injury	
yes	0 (0.0%)
no	8 (100.0%)
Facial nerve management	
decompression	7 (87.5%)
graft	0 (0.0%)
Anastomosis (combined with rerouting)	1 (12.5%)
Postoperative grade (H-B)	
I	2 (25.0%)
II	4 (50.0%)
III	2 (25.0%)

^a^Five patients did not meet the inclusion criteria of this study

^b^Site of facial nerve injury: one (12.5%) patient suffered from multisegment injury

For 37 patients transferred, eighteen (48.6%) were male and 19 (51.4%) were female. Median age was 35.1 ± 15.0 (range 7–62) years old. Twenty-one (56.8%) patients paralyzed on the right and 16 (43.2%) on the left. Four (8.9%) patients had middle ear surgery history before primary surgery. All the 37 patients received corticosteroid and antibiotics after paralysis; in addition, four (8.9%) patients had also received exploration before transferring to our center for re-revision surgery due to unsatisfactory recovery (Table 2 and Supplementary Table 1).

**Table 2:** Analysis of 37 patients transferred from other centers

Clinical characteristics	
Gender	
male	18 (48.6%)
female	19 (51.4%)
Age (years)	35.1 ± 15.0 (7–62)
Side	
right	21 (56.8%)
left	16 (43.2%)
Preoperative grade (H-B)	
V	13 (35.1%)
VI	24 (64.9%)
Preoperative hearing^a^	
complete deafness	18 (48.6%)
conductive hearing loss	10 (27.0%)
mixed hearing loss	9 (24.3%)
Duration before revision surgery	
< 2 months	19 (51.4%)
2–6 months	10 (27.0%)
> 6 months	8 (21.6%)
Site of facial nerve injury^b^	
tympanic segment	27 (73.0%)
second genu	24 (64.9%)
mastoid segment	8 (21.6%)
geniculate ganglion	4 (10.8%)
Degree of facial nerve injury^c^	
edema	7 (18.9%)
sheath defective and edema	5 (13.5%)
superficially transected	2 (5.4%)
partially transected	4 (10.8%)
completely transected	19 (51.4%)
Malformation of facial nerve	
anomalous course	1 (2.7%)
bifurcation	1 (2.7%)
Inner ear injury	
yes	18 (48.6%)
no	19 (51.4%)
Facial nerve management	
decompression (2 cases combined with axion realignment)	14 (37.8%)
graft	19 (51.4%)
Anastomosis	4 (10.8%)
Postoperative grade (H-B)	
I	6 (16.2%)
II	6 (16.2%)
III	18 (48.6%)
IV	5 (13.5%)
V	2 (5.4%)

^a^Preoperative hearing: according to 2021 WHO classification of hearing loss

^b^Site of facial nerve injury: 21 (56.8%) patients suffered from multisegment injury

^c^Degree of facial nerve injury: superficially transected (< 1/3 of the circumference), partially transected (> 1/3 of the circumference)

### Preoperative H-B grade and hearing

Before revision surgery, for 8 patients injured at our center, seven (87.5%) ranked H-B grade V and one (12.5%) ranked H-B VI, three (37.5%) suffered from conductive hearing loss and five (62.5%) mixed hearing loss. For 37 patients transferred, thirteen (35.1%) ranked H-B grade V and 24 (64.9%) ranked H-B VI, eighteen (48.6%) suffered from complete deafness, ten (27.0%) conductive hearing loss and nine (24.3%) mixed hearing loss. All outcomes of EMG exam indicated spontaneous fibrillation potential and (or) loss of active activity of motor unit, and only 7 patients were found suitable for concurrent ossicular chain reconstruction.

### Site of facial nerve injury

**Fig. 1:**
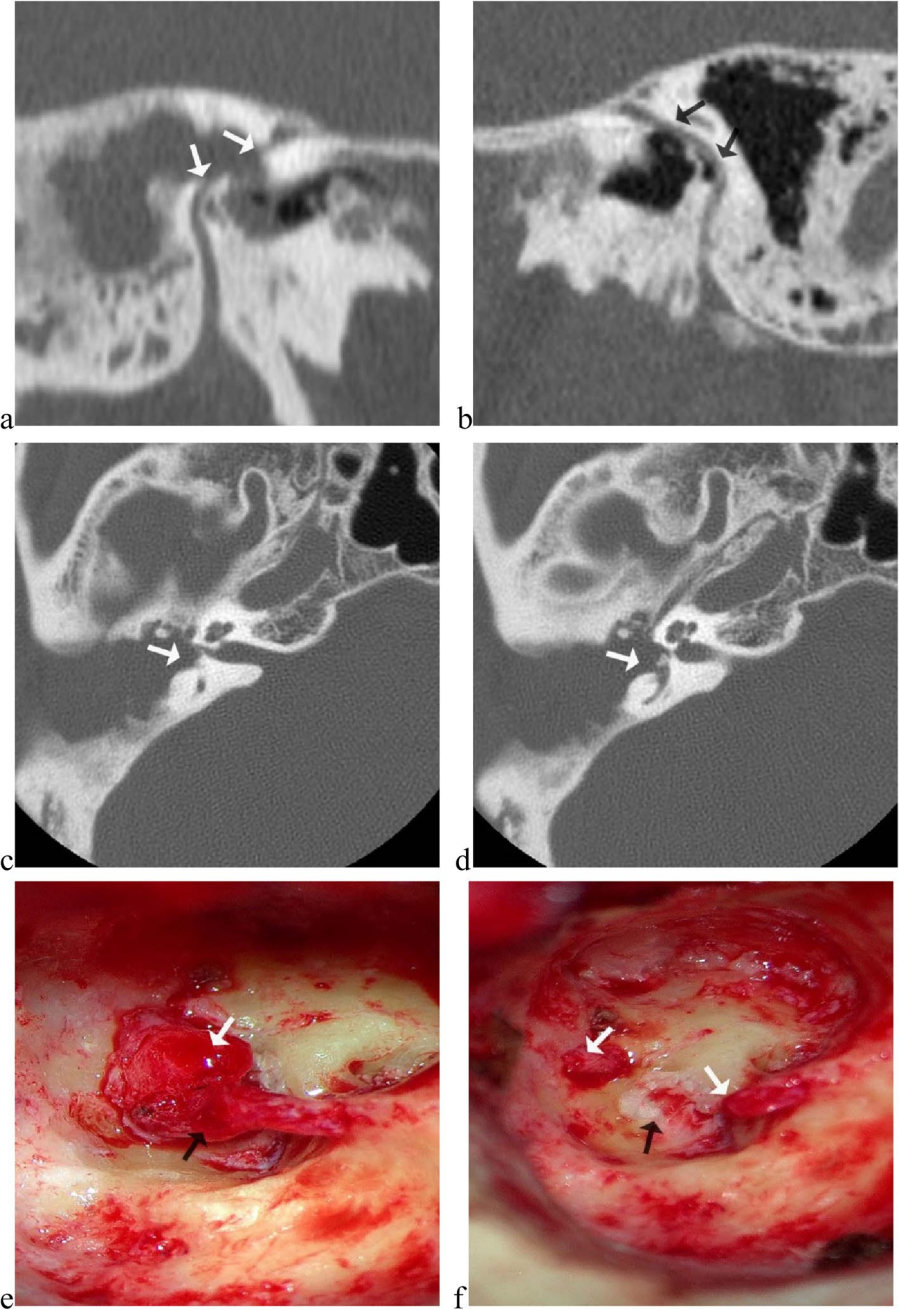
Determination of the injured site of facial nerve by preoperative high-resolution computed tomography (HRCT) and inoperative exploration. Illustrated case, male, 51-year-old, suffered from complete transection of facial nerve between the second genu and tympanic segment in the tympanoplasty for right-side chronic suppurative otitis media. **a** Multiplanar reconstructed CT image revealed the fallopian canal of the second genu and tympanic segment was defective on the right; **b** on the contralateral left side, the whole course of fallopian canal was intact; **c d** axial CT images revealed fallopian canal of tympanic segment was defective, and vestibule was injured; **e** correspondingly in exploration, the fallopian canal of the second genu and tympanic segment was defective, the facial nerve was transected (black arrow) and traumatic neuroma developed at the proximal ending (white arrow); **f** after removal of the traumatic neuroma and trimming the endings (white arrow), vestibule was found injured (black arrow)

**Fig. 2:**
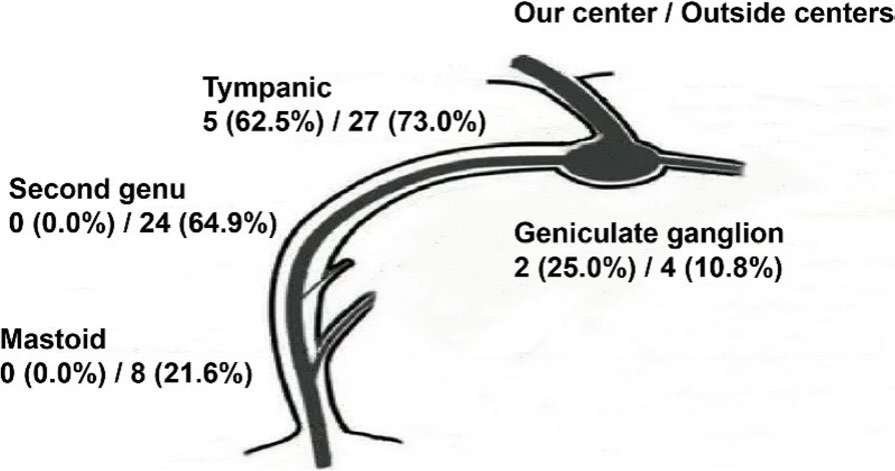
Site of facial nerve injury at our center and outside centers. For 8 patients injured at our center, tympanic segment injury was found in 5 (62.5%) cases, geniculate ganglion injury in 2 (25.0%) cases, and fallopian canal intact in 2 (25.0%) cases; besides, one (12.5%) patient suffered from multisegment injury. For 37 patients transferred, tympanic segment and second genu were the most common injury site, accounted for 27 (73.0%) cases and 24 (64.9%) cases respectively, mastoid segment and geniculate ganglion injury accounted for 8 (21.6%) cases and 4 (10.8%) cases, and 21 (56.8%) patients suffered from multisegment injury

Site of facial nerve injury was determined by preoperative HRCT and inoperative exploration (Fig. 1). For 8 patients injured at our center, tympanic segment injury was found in 5 (62.5%) cases, geniculate ganglion injury in 2 (25.0%) cases, and fallopian canal intact in 2 (25.0%) cases; besides, one (12.5%) patient suffered from multisegment injury. For 37 patients transferred, tympanic segment and second genu were the most common injury site, accounted for 27 (73.0%) cases and 24 (64.9%) cases respectively, mastoid segment and geniculate ganglion injury accounted for 8 (21.6%) cases and 4 (10.8%) cases, and 21 (56.8%) patients suffered from multisegment injury (Fig. 2).

### Degree of facial nerve injury and management

Degree of facial nerve injury varied. For 8 patients injured at our center, seven (87.5%) patients suffered from mild injury, with fallopian canal intact and facial nerve mild edematous 2 (25.0%) cases, facial nerve edematous 4 (50.0%) cases, sheath defective and nerve edematous 1 (12.5%) case; besides, nerve completely transected in 1 (12.5%) case; For 37 patients transferred, seven (18.9%) patients suffered from nerve edema, five (13.5%) sheath defective and edema, two (5.4%) superficially transected, four (10.8%) partially transected, and 19 (51.4%) completely transected, with 10 (27.0%) developing traumatic neuroma.

**Fig. 3:**
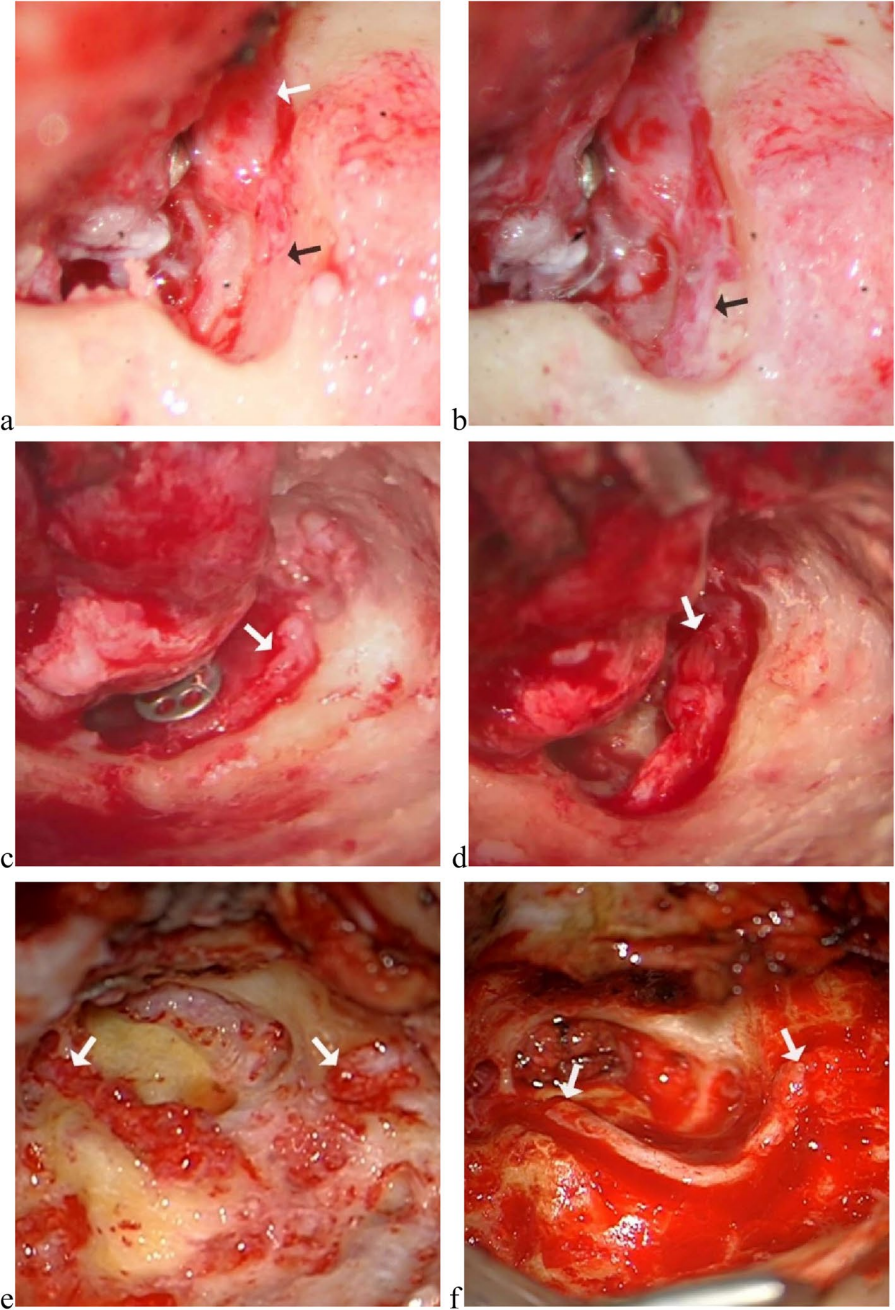
Different degrees of facial nerve injury and corresponding management. **a b** Illustrations of the decompression; **a** facial nerve was edematous (white arrow) and fallopian canal was defective (black arrow); **b** drilled the fallopian canal until more than 5 mm normal neural trunk appeared. **c d** Illustrations of decompression and realignment of axons; **c** facial nerve was superficially transected and edematous; **d** decompressed facial nerve, realigned the axons and reset the sheath. **e f** Illustrations of great auricular nerve graft; **e** facial nerve was transected between geniculate ganglion and mastoid segment; **f** the great auricular nerve graft was interposed between transected nerve endings

Different degrees of facial nerve injury received different surgical management (Fig. 3). Decompression (21, 46.7%) was conducted for 11 cases with fallopian canal defective and facial nerve edematous, 6 cases with sheath defective and facial nerve edematous, and decompression combined with realignment of axons was conducted for 2 cases with facial nerve superficially transected; for 2 cases with fallopian canal intact, decompression from stylomastoid foramen to geniculate ganglion was conducted and facial nerve was found mildly edematous. Great auricular nerve graft (19, 42.2%) was conducted for 19 cases with facial nerve completely transected. Anastomosis (5, 11.1%) was performed for 4 cases partially transected (direct anastomosis) and 1 case with facial nerve completely transected (rerouting and end-to-end tension-free anastomosis).

### Other findings in revision surgery

Malformation of facial nerve was found in 8 (17.8%) cases of the total 45 patients. For 8 patients injured at our center, fallopian canal dehiscent was found in 5 (62.5%) cases, anomalous course in 1 (12.5%) case. For 37 patients transferred, anomalous course and bifurcation accounted for 1 (2.7%) case respectively (fallopian canal dehiscent in revision surgery was not calculated for difficulty in identifying the presence before primary surgery); besides, residual lesions were also found in 14 (37.8%) cases and inner ear injury in 18 (48.6%) cases.

### Final postoperative H-B grade and prognostic factor

**Fig. 4:**
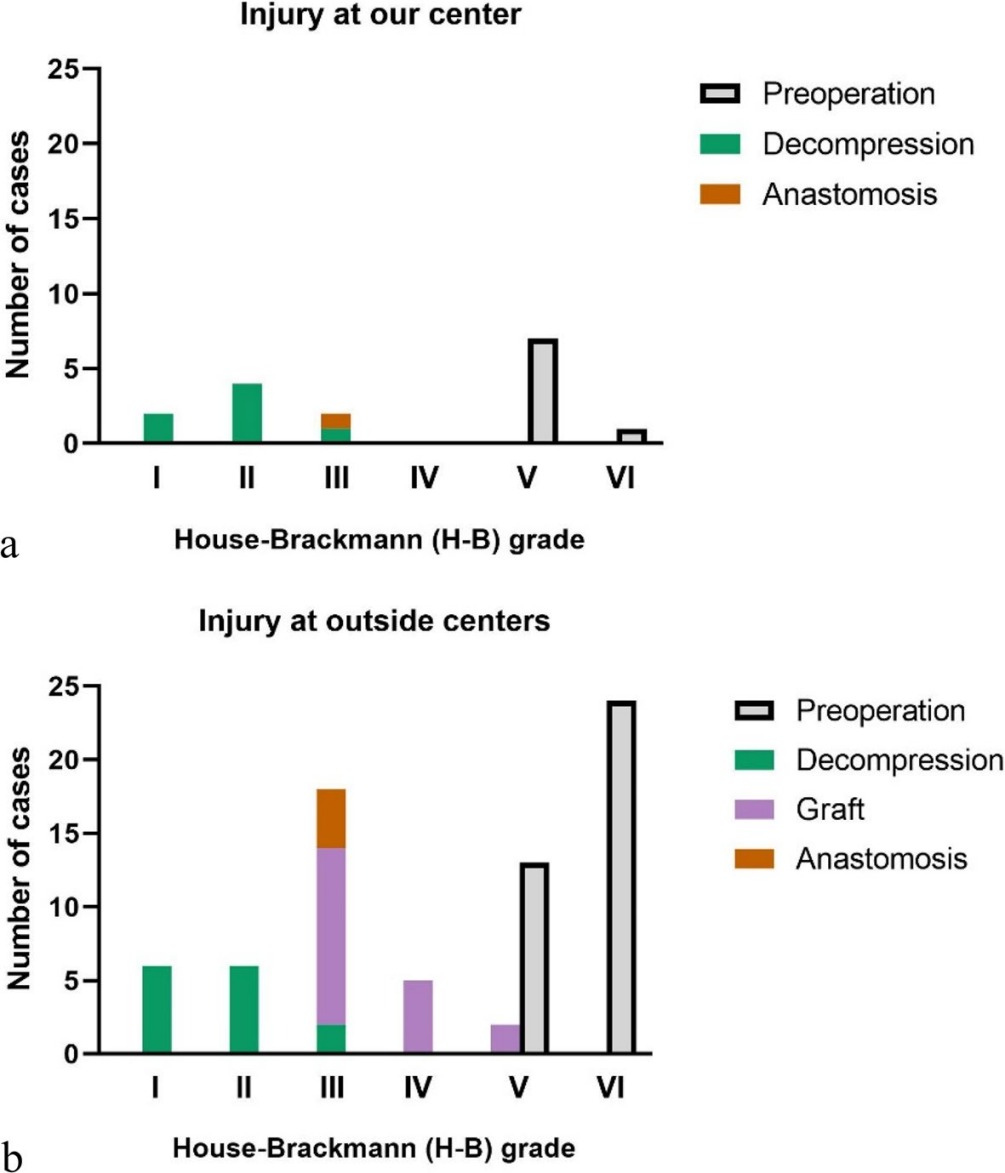
Preoperative and postoperative House-Brackmann (H-B) grade of 8 cases injured at our center and 37 cases transferred. **a** For 8 patients injured at our center, seven (87.5%) ranked H-B grade V and one (12.5%) ranked H-B VI before revision surgery; seven (87.5%) received decompression and one (12.5%) received anastomosis; two (25.0%) recovered to H-B grade I, four (50.0%) recovered to H-B II, and the other two (25.0%) recovered to H-B III. **b** For 37 patients transferred, thirteen (35.1%) ranked H-B grade V and 24 (64.9%) ranked H-B VI preoperatively; fourteen (37.8%) received decompression, nineteen (51.4%) received graft and one (12.5%) received anastomosis; final postoperative grade ranked from H-B grade I to grade V, with H-B I 6 (16.2%) cases, H-B II 6 (16.2%) cases, H-B III 18 (48.6%) cases, H-B IV 5 (13.5%) cases and H-B V 2 (5.4%) cases

All the patients were followed up for more than 2 years. For 8 patients injured at our center, seven (87.5%) received decompression and one (12.5%) received anastomosis; two (25.0%) patients recovered to H-B grade I, four (50.0%) recovered to H-B II, and the other two (25.0%) recovered to H-B III. For 37 patients transferred, fourteen (37.8%) received decompression, nineteen (51.4%) received graft and one (12.5%) received anastomosis; final postoperative grade ranked from H-B grade I to grade V, with H-B I 6 (16.2%) cases, H-B II 6 (16.2%) cases, H-B III 18 (48.6%) cases, H-B IV 5 (13.5%) cases and H-B V 2 (5.4%) cases (Fig. 4).

**Fig. 5:**
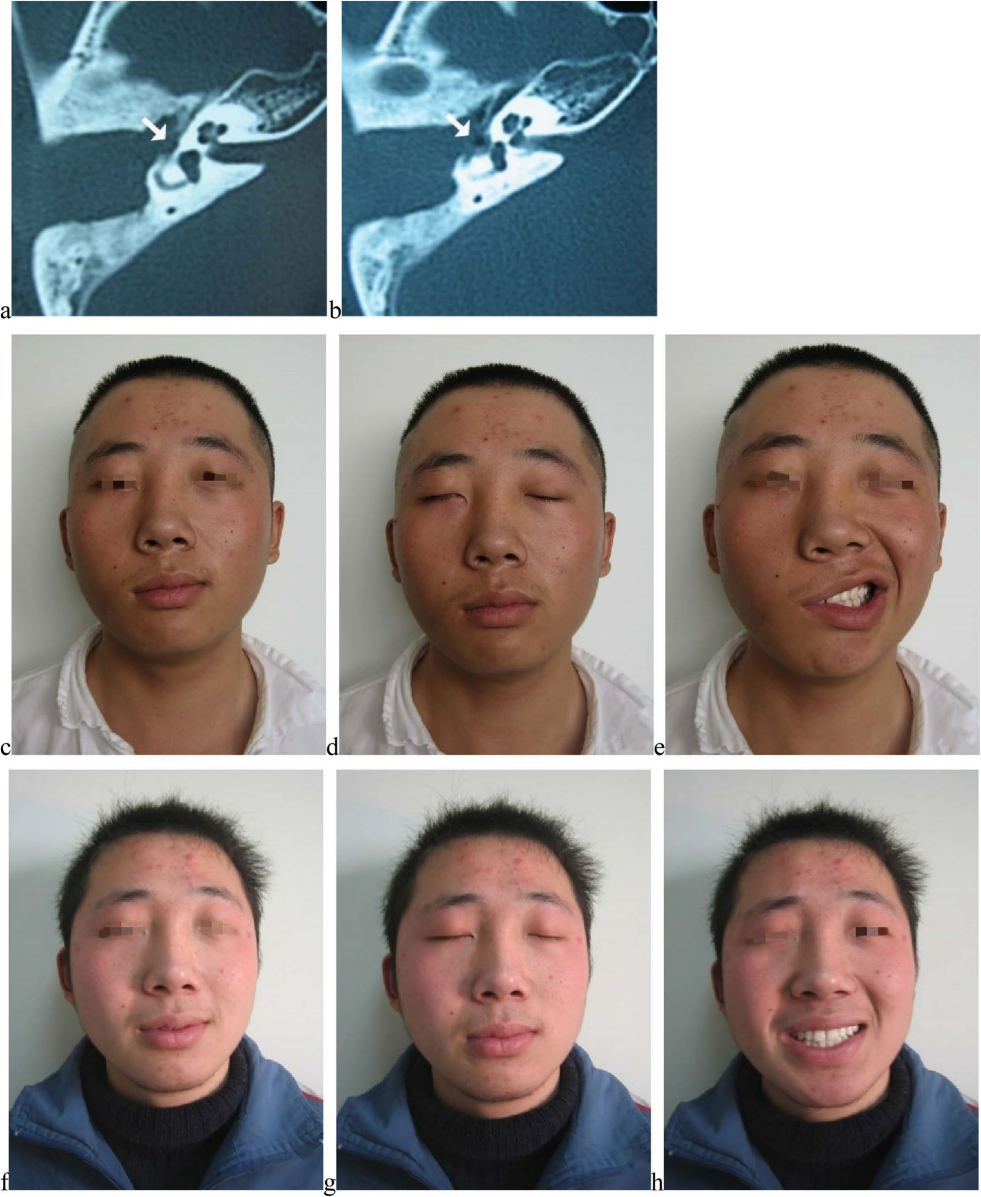
Recovery from H-B grade V to H-B grade I after decompression. No. 12 patient, male, 17-year-old, suffered from facial nerve edematous in the canal wall down mastoidectomy for right-side cholesteatoma of middle ear, received decompression within 2 months. **a b** Axial CT images revealed fallopian canal of tympanic segment was defective. **c d e** Facial images before decompression; **c** at rest before decompression; **d** eye closure before decompression; **e** smile before decompression. **f g h** Facial images after decompression; **f** at rest after decompression; **g** eye closure after decompression; **h** smile after decompression

For decompression, before surgery, twenty (95.2%) patients ranked H-B grade V and 1 (4.8%) patient ranked H-B VI. After surgery, majority recovered to H-B I (8, 38.1%) and H-B II (10, 47.6%), only 3 (14.3%) patients recovered to H-B III (one was decompressed over 6 months after paralysis, one with facial nerve superficially transected and the other one with congenital H-B II paralysis) (Fig. 5). Patients received decompression within 2 months after paralysis had higher possibility of H-B I or II recovery than those over 2 months (*P* = 0.026), and of 8 patients who recovered to H-B I, majority (6, 75.0%) were also decompressed within 2 months. Recovery could start as early as within 1 month after decompression, and most patients (more than 80.0%) could gain the maximum recovery within 6 months (Table 3).

**Table 3:** Analysis of 45 patients’ clinical characteristics

Clinical characteristics		
	H-B grade I and II recovery/H-B grade III recovery	*P* = 0.026
Decompression within 2 months	14/0	
Decompression over 2 months	4/3	
	H-B grade III recovery/H-B grade IV and V recovery	*P* = 0.041
Graft within 6 months	10/3	
Graft over 6 months	1/5	
	H-B grade III recovery/H-B grade IV and V recovery	*P* = 0.575
Anastomosis within 6 months	5/0	
Graft within 6 months	11/2	
	Exploration before revision surgery^a^ (yes/no)	*P* = 0.001
H-B grade I or II recovery of decompression, H-B grade III recovery of graft	0/31	
H-B grade III recovery of decompression, H-B grade IV or V recovery of graft	4/5	

^a^Exploration before revision surgery: One patient received decompression and three patients received graft before transferred

**Fig. 6:**
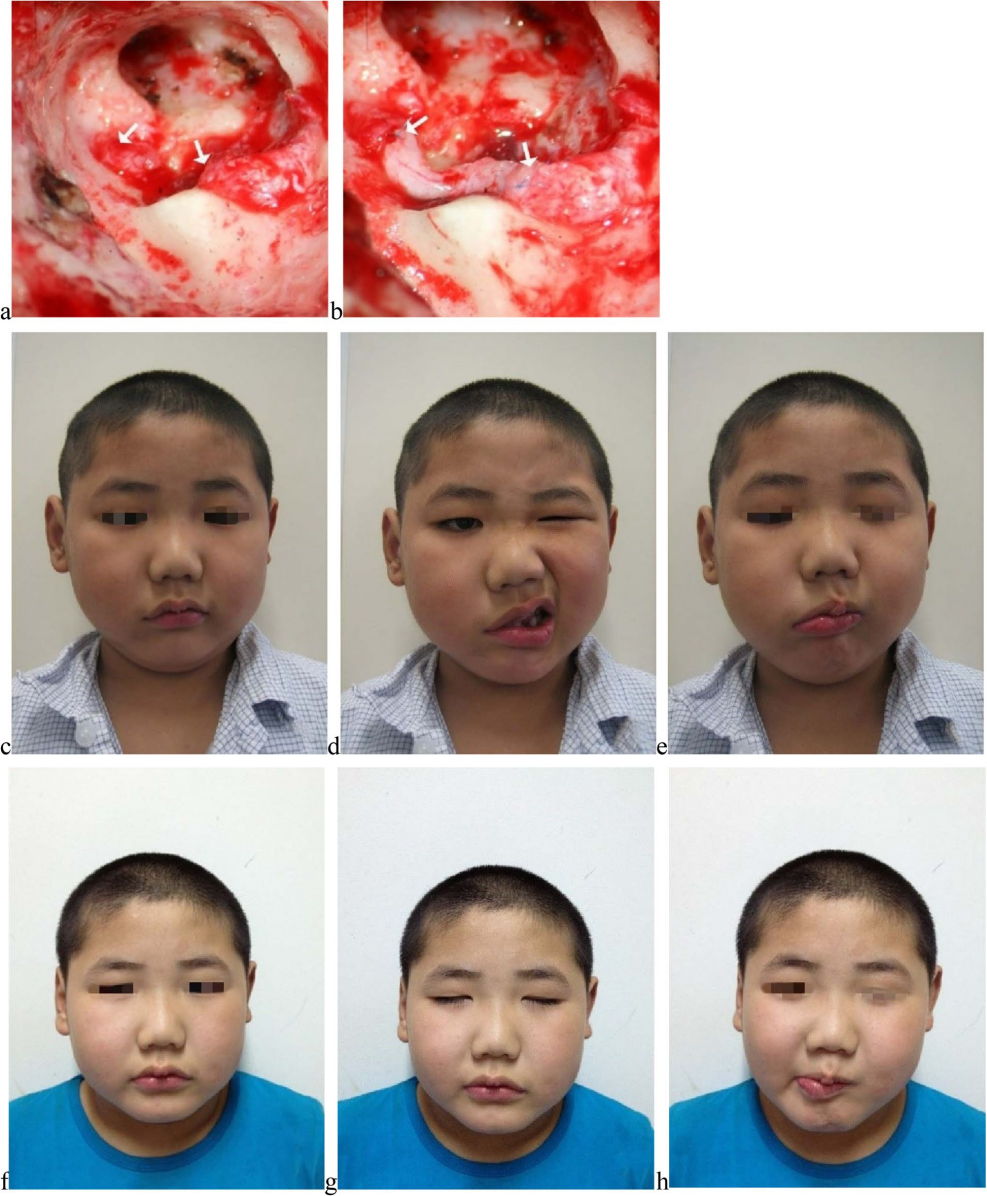
Recovery from H-B grade VI to H-B grade III after great auricular nerve graft. No. 39 patient, male, 7-year-old, suffered from complete facial nerve transection in surgery for right-side chronic suppurative otitis media, received graft within 12 days. **a b** Illustrations of great auricular nerve graft. **c d e** Facial images before graft; **c** at rest before graft; **d** eye closure before graft; **e** upper and lower lip movement before graft. **f g h** Facial images after graft; **f** at rest after graft; **g** eye closure after graft; **h** upper and lower lip movement after graft

For graft, before surgery, all the patients ranked H-B grade VI. After surgery, the best recovery was H-B III (12, 63.2%) (Fig. 6). Besides, 5 (26.3%) patients recovered to H-B IV, and 2 patients (10.5%) only recovered to H-B V (both received graft over 6 months after paralysis). Patients received graft within 6 months were more likely to get H-B III recovery (*P* = 0.041). For recovery, although there was patient found first sign of reinnervation 3 months after surgery, most (75.0%) reported reinnervation started around 6 months after graft, and continued up for over 1 year or even to 2 years.

**Fig. 7:**
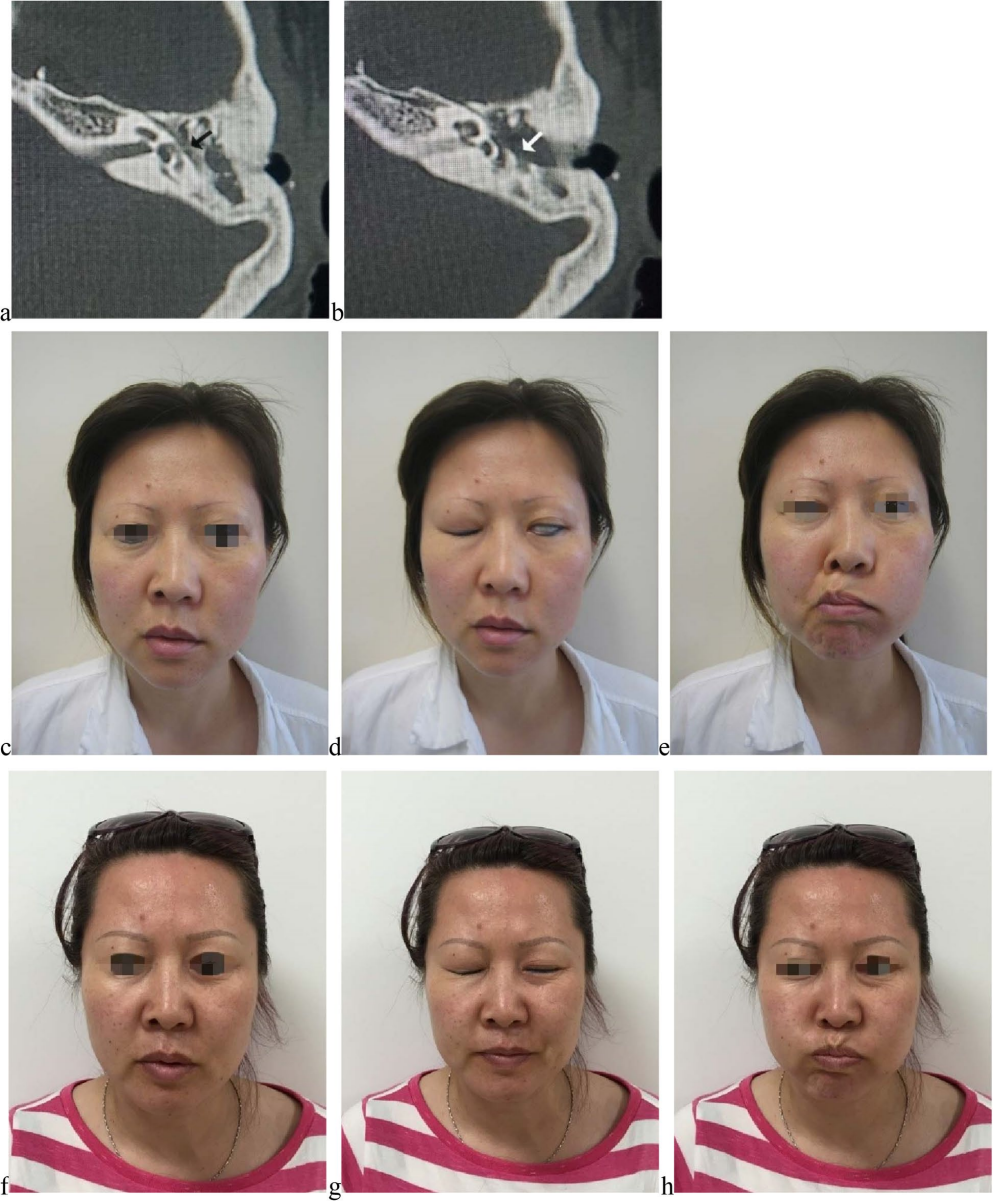
Recovery from H-B grade VI to H-B grade III after direct anastomosis. No. 18 patient, female, 37-year-old, suffered from more than 1/3 facial nerve transection in the canal wall down mastoidectomy for left-side cholesteatoma of middle ear, received direct anastomosis within 25 days. **a b** Axial CT images revealed fallopian canal of tympanic segment and the second genu were defective. **c d e** Facial images before graft; **c** at rest before anastomosis; **d** eye closure before anastomosis; **e** upper and lower lip movement before anastomosis. **f g h** Facial images after anastomosis; **f** at rest after anastomosis; **g** eye closure after anastomosis; **h** upper and lower lip movement after anastomosis

For anastomosis, all (5, 100.0%) the patients ranked H-B grade VI preoperatively. All of them underwent anastomosis within 6 months after paralysis and all recovered to H-B III (Fig. 7). Although no patient recovered to worse than H-B III, there was no statistical significance (*P* = 0.575) between H-B III recovery of anastomosis and graft when they were both conducted within 6 months after paralysis. For recovery, one patient reported reinnervation could start as early as 3 months after surgery, but majority (60.0%) reported recovery lasted from 6 months to 1 year or longer.

In addition, four (8.9%) patients had received exploration before transferred to our center for re-revision surgery. Neither of them gained H-B grade I or II from decompression, or H-B grade III from graft (no anastomosis was conducted). Statistical analysis showed that they gained poorer recovery than those without previous revision surgery at nonacademic centers (*P* = 0.001).

## Discussion

Being one of the severest complications of middle ear surgery, iatrogenic facial nerve paralysis has always been the accident that every otologist tries his best to avoid. Fortunately, the incidence has declined. In our center, it has dropped from 0.13% (7/5491) to 0.04% (6/14552) over the past 2 decades. Worldwide, it was reported between 0.6% and 3.6% [4, 5] last century, and is assumed to be lower than 0.10% [3] at present. One reason contributing to this is the application of advanced surgical instruments, such as facial nerve monitor and high-definition microscope. However, iatrogenic facial nerve paralysis still happens now and then. When iatrogenic facial nerve paralysis happens anyway, knowing how to properly manage it will be beneficial to the recovery, that is the reason why we carry out this study.

### Primary disease and surgery

For the injury happened at our center, most happened in the surgery for cholesteatoma of middle ear (4, 50.0%), because surgery for cholesteatoma was often more complicated than that of other middle ear disease [6]. Besides, most surgeries (37, 82.2%) and severe injury (partial or complete transection, 23, 95.8%) were conducted at outside nonacademic centers, lack of clinical experience and adequate temporal bone dissection training may contribute to the facial nerve injury.

### Risk factors

Malformation of facial nerve. Dehiscence of fallopian canal is the most common high-risk factor; it may be congenital or eroded by lesion. The incidence was reported as high as 56% in an anatomy of 1000 temporal bones [7] and 30%-33% in cholesteatoma surgeries [8, 9]. In our study, the incidence was 62.5% in the cases injured at our center. The risk of dehiscence comes from exposure of facial nerve directly to drills, sharp surgical instruments, or even some irritating substances [10], and preoperative HRCT could help identify it. In addition, deformed facial nerve would make localization and identification difficult. In our study, anomalous course and bifurcation of facial nerve were also identified. History of congenital facial nerve paralysis is uncommon but may add risk to idiopathic facial nerve paralysis. One patient (No. 44 case in Supplementary Table 1) with congenital H-B grade II paralysis suffered from H-B grade V postoperative paralysis; during exploration, fallopian canal was intact and facial nerve was only mildly edematous, we assumed it may be increased nerve susceptibility.

Revision surgery. During revision surgery, localization of the facial nerve becomes difficult due to removed landmarks and fibrosis. In our study, four (8.9%) cases of injury happened in revision surgery.

Misdiagnosis. Some facial nerve tumors are difficult to distinguish from cholesteatoma or granulation tissues [11] (our cases were excluded by exclusion criteria), once misdiagnosis is done before surgery, facial nerve paralysis is very likely to happen. It is crucial an otologist bore in mind the lesion on the surface of facial nerve might be the tumor originates from the nerve trunk, although preoperative facial nerve function could be normal.

### Common sites of facial nerve injury

The most venerable site was tympanic segment (32, 71.1%), and many patients (22, 48.9%) suffered from multisegment injury, which were consistent with other report [12]. In cholesteatoma and chronic otitis media surgery, lesions are often near tympanic sinus, so distal tympanic segment, and sometimes the second genu are easy to be injured. The tympanic segment near oval window is prone to be injured when lesions locate near stape, especially when fallopian canal is dehiscent [13]. The geniculate ganglion should be attention to when clearing the lesions near the cochleariform process [14]. The injury of labyrinthine segment is rare; however, when clearing cholesteatoma above tympanic segment in the attic, labyrinthine segment is at high risk because facial nerve turns backward and inward from geniculate ganglion.

### Degree of facial nerve injury and preventive measures

Mild injury normally only leads to nerve edema. In our study, 28.8% patients suffered from such injury. Edema could result from retraction of nerve, to avoid it, the lesions should be cleared along the nerve trunk. It could also come from compression by bone chips or hematoma, so careful examination and hemostasis before the end of surgery was important. Heat from drills, electrical stimulation from coagulator lead to thermal damage and nerve edema, so timely rinse is important to taking away the heat. Notably, two cases with canal intact were also found facial nerve mildly edematous after decompression. One of them suffered from congenital H-B grade II paralysis, we assumed it may be increased nerve susceptibility; the other one may suffered from herpes simplex virus reactivation, which was consistent with the pathology of Bell palsy [13, 15]; for there was no direct injury to the facial nerve, they might have recovered without exploration. In addition, prognosis of facial nerve with sheath defective is nearly as good as with edema because the axons remain intact. In our study, 13.3% patients suffered from sheath defective and none recovered to worse than H-B II. This kind of injury often comes from accident bruise in an unclear operative field, so try to rinse and identify the facial nerve, nerve is unlikely to be injured when it has been identified [16]. When the nerve cannot be distinguished from lesions, the facial nerve monitor can help, the current should not be excessive but be gradually adjusted to the threshold. What is more, facial nerve monitor cannot take the place of proficient anatomical knowledge and rich clinical experience despite its wide application.

The severe injury is direct transection, which accounts for 57.8% cases in our study. To avoid it, the movement of high-speed drill or micro cutting equipment should be along the course of facial nerve and appropriate sizes should be chosen for different lesions. Besides, half patients with nerve transection developed neuroma, more than 90% happened over 3 months after paralysis. Before nerve repair, traumatic neuroma has to be removed, which sacrifices the length of facial nerve and potentially makes direct anastomosis unavailable, so early exploration could reduce the possibility of autologous nerve graft.

### Determination of exploration and management

Immediate and severe paralysis (H-B grade V or VI) should receive more attention, because the earlier and severer a paralysis, the more likely the nerve suffers from substantial damage. However, tight dressings and packing should be released and time allowed for any local anesthetic effects to dissipate [14], and some patients can still close eyes upon forceful eyelid closure even a few hours after a complete transection of facial nerve [3]. Once immediate and severe paralysis is diagnosed, whether exploration be conducted should be further weighed. If the otologist is certain of nerve integrity, such as the whole course of the facial nerve was identified in surgery or the fallopian canal remained intact, then a conservative approach can be adopted. When there are some uncertainties, HRCT scan could help identify the integrity of fallopian canal. If fallopian canal is not integral, exploration should be conducted at the earliest time, if integral, conservative treatment could be tried. However, if HRCT indicates no obvious injury but there is no sign of clinical and electrophysiological improvement after conservative treatment, exploration should also be conducted timely. In our study, all the patients suffered from immediate and severe paralysis, with no sign of improvement after conservative treatment, and no otologist was certain of nerve integrity. During exploration, a general rule is decompression for mild injury, re-approximation of axions for superficial transection, anastomosis for partial or complete transection, and graft is adopted when end-to-end tension-free anastomosis (even with rerouting) is not applicable.

### Prognostic factors

The severity of facial nerve injury. The milder the injury, the better the outcome. Most of patients (85.7%) with mild injury recovered to H-B grade I and II, and none recovered to worse than H-B III. On the contrary, for those with facial nerve partially or completely transected, the best recovery was H-B III. The prognosis of anastomosis seemed better than graft because no patient recovered to worse than H-B III, but statistical analysis did not show significance (*P* = 0.575). Maybe it was due to limited sample size, or there was just no difference between them [17], in the future a larger series is needed to validate it.

Duration before revision surgery. For decompression, those received surgery within 2 months after paralysis had higher possibility of H-B grade I or II recovery than those over 2 months (*P* = 0.026), and for patients recovered to H-B I, seventy-five percent were also decompressed within 2 months. Prolonged edema or compression was found to reduce the probability of a satisfactory recovery [18, 19]. For graft, those received surgery within 6 months were more likely to get H-B III recovery (*P* = 0.041). This is supported by the theory that nerves regenerate at about 1 mm/day [20], and muscles undergo irreversible atrophy from 12 months after denervation [21], considering the distance for nerve regeneration from sectioned ending to distal motor endplate, graft had better been conducted within 6 months other than 12 months as previously recommended [22, 23]. As to anastomosis, all patients in our study were conducted within 6 months, and all recovered to H-B III.

Re-revision surgery. Four patients received exploration at nonacademic centers before transferring to our center for re-revision surgery, they all gained worse recovery than those without previous exploration (*P* = 0.001). The main reason may be additional surgical trauma from nonstandard revision surgical procedure and consequent delayed re-revision surgery.

## Conclusion

Cholesteatoma of middle ear is a common primary disease that leads to iatrogenic facial nerve injury. High-risk factors include facial nerve malformation, revision surgery and misdiagnosis. Tympanic segment is the common site of facial nerve injury. If iatrogenic facial nerve paralysis happens after surgery, HRCT could help identify the injured site. The severity of facial nerve injury, duration before revision surgery and re-revision surgery are prognostic factors. Decompression conducted within 2 months after paralysis, graft and anastomosis conducted within 6 months after paralysis lead to better recovery.

## Supplementary Information

**Additional file 1:** **Supplementary Table 1.** Clinical characteristics of 45 patients.

## Data Availability

All data generated or analyzed during this study are included in this published article.

## Abbreviations

H-B: House-Brackmann
HRCT: High-resolution computed tomography
EMG: Electromyogram
