# Supplementary material for: Surgical management and the prognosis of iatrogenic facial nerve injury in middle ear surgery: a 20-year experience
Source: Head Face Med. 2023 Jul 25;19:31. doi: 10.1186/s13005-023-00377-y (PMC10369826; doi:10.1186/s13005-023-00377-y)
Supplement: Supplementary file 1 — Additional file 1: Supplementary Table 1. Clinical characteristics of 45 patients. [file 13005_2023_377_MOESM1_ESM.docx]

**Supplementary Table 1. Clinical Characteristics of 45 Patients**

| **No.** | **Gender** | **Age (years)** | **Side** | **Center ^a^** | **Primary disease ^b^** | **Type of primary** **surgery ^c^** | **Preoperative grade (H-B) ^d^** | **Preoperative hearing ^e^** | **Duration before surgery**  **(Days or Months) ^f^** | **Site of facial nerve injury ^g^** | **Degree of facial nerve injury ^h^** | **Malformation of facial nerve** | **Inner ear injury (Yes/No)** | **Facial nerve management** | **Final postoperative grade (H-B) ^i^** |
| --- | --- | --- | --- | --- | --- | --- | --- | --- | --- | --- | --- | --- | --- | --- | --- |
| 1 | Female | 36 | Right | Other | Chol | CWD | 5 | CHL | 32D | Tymp | Sheath defective, Edema | None | N | Decompression | 2 |
| 2 | Female | 41 | Right | Other | Chol | CWD | 5 | CHL* | 45D | Sg, Mast | Edema | None | N | Decompression | 1 |
| 3 | Female | 55 | Right | Other | Chol | CWU | 6 | MHL** | 3M+ | Sg | Partially transected | None | N | Anastomosis | 3 |
| 4 | Female | 23 | Right | Other | Chol | CWD | 5 | CHL | 19D | Sg | Sheath defective, Edema | None | N | Decompression | 1 |
| 5 | Female | 52 | Right | Other | Chol | CWD | 6 | MHL | 5M+ | Tymp | Completely transected* | None | N | Graft | 3 |
| 6 | Female | 48 | Right | Our | Chol | CWU* | 5 | CHL | 9D | Tymp | Edema | None | N | Decompression | 2 |
| 7 | Male | 13 | Right | Other | Chol | CWD | 5 | CHL | 60D | Tymp | Superficially transected | Bifurcation | N | Decompression, Realignment | 3 |
| 8 | Female | 18 | Right | Our | Chol | CWD | 6 | MHL | 46D | Tymp | Completely transected | Canal dehiscent | N | Anastomosis, Rerouting | 3 |
| 9 | Female | 31 | Right | Our | Chol | CWD | 5 | CHL | 9D | GG | Edema | Canal dehiscent | N | Decompression | 2 |
| 10 | Female | 11 | Right | Other | Chol | CWD | 5 | MHL | 7M+ | Tymp, Sg, Mast | Sheath defective, Edema | None | N | Decompression | 2 |
| 11 | Male | 60 | Right | Other | Chol | CWD* | 6 | CD | 8M+ | GG, Tymp | Completely transected* | None | Y | Graft | 5 |
| 12 | Male | 17 | Right | Other | Chol | CWD | 5 | CHL | 57D | GG, Tymp, Sg | Sheath defective, Edema | None | N | Decompression | 1 |
| 13 | Female | 43 | Left | Our | Chol | CWD | 5 | MHL* | 54D | GG, Tymp | Sheath defective, Edema | Canal dehiscent | N | Decompression | 2 |
| 14 | Female | 15 | Right | Other | Chol | CWD | 6 | CD | 51D | Tymp, Sg | Completely transected* | None | Y | Graft | 3 |
| 15 | Male | 32 | Left | Other | Chol | CWD | 6 | MHL | 5M+ | Sg | Partially transected | Canal dehiscent | N | Anastomosis | 3 |
| 16 | Female | 46 | Right | Other | Chol | CWD | 6 | CD | 6M+ | Tymp, Sg | Completely transected* | None | Y | Graft | 3 |
| 17 | Female | 49 | Left | Other | Chol | CWD | 6 | CD | 34D | Tymp, Sg | Completely transected | None | Y | Graft | 4 |
| 18 | Female | 37 | Left | Other | Chol | CWD | 6 | CD | 25D | Tymp, Sg | Partially transected | None | Y | Anastomosis | 3 |
| 19 | Female | 43 | Right | Other | Chol | CWD | 5 | CD | 3M+ | Tymp, Sg | Edema | None | Y | Decompression | 1 |
| 20 | Male | 29 | Left | Other | Chol | CWD | 5 | CHL | 37D | Tymp | Edema | None | N | Decompression | 2 |
| 21 | Male | 59 | Right | Other | Chol | CWD | 6 | CD | 7M+ | Tymp | Completely transected* | None | Y | Graft | 4 |
| 22 | Male | 27 | Right | Other | Chol | CWD | 6 | MHL | 8D | Sg | Completely transected | None | N | Graft | 3 |
| 23 | Female | 42 | Left | Other | Chol | CWD | 6 | CD | 4M+ | Sg, Mast | Completely transected* | None | Y | Graft | 3 |
| 24 | Male | 19 | Right | Other | Chol | CWD | 5 | CD | 3M+ | GG, Tymp | Edema | None | Y | Decompression | 2 |
| 25 | Male | 20 | Left | Other | Chol | CWD | 6 | CD | 3M+ | Tymp, Sg | Completely transected | None | Y | Graft | 3 |
| 26 | Female | 20 | Left | Other | Chol | CWD* | 6* | CD | 7M+ | Tymp, Sg, Mast | Completely transected* | None | Y | Graft | 4 |
| 27 | Male | 50 | Left | Other | CSOM | Tympanoplasty* | 5 | CD | 21D | Tymp | Superficially transected | None | Y | Decompression, Realignment | 2 |
| 28 | Male | 32 | Right | Other | CSOM | Tympanoplasty | 6 | MHL | 17D | Tymp, Sg | Partially transected | None | N | Anastomosis | 3 |
| 29 | Male | 41 | Right | Other | CSOM | CWD | 6 | CD | 3M+ | Sg, Mast | Completely transected | None | Y | Graft | 3 |
| 30 | Male | 28 | Left | Other | CSOM | Tympanoplasty | 6 | CHL | 41D | Tymp, Sg, Mast | Completely transected | None | N | Graft | 3 |
| 31 | Male | 51 | Left | Other | CSOM | CWD | 6 | CD | 6M+ | Tymp | Completely transected* | None | Y | Graft | 4 |
| 32 | Male | 53 | Right | Our | CSOM | Tympanoplasty | 5 | MHL** | 2M+ | / | Canal intact, Mild edema | None | N | Decompression | 1 |
| 33 | Male | 62 | Left | Other | CSOM | CWD | 6 | CD | 40D | Tymp, Sg | Completely transected | None | Y | Graft | 3 |
| 34 | Male | 43 | Left | Other | CSOM | Tympanoplasty | 6 | CD | 7D | Tymp | Completely transected | None | Y | Graft | 3 |
| 35 | Female | 49 | Left | Other | CSOM | CWD | 6 | CHL | 11D | GG, Tymp | Sheath defective, Edema | None | N | Decompression | 2 |
| 36 | Female | 47 | Left | Our | CSOM | Tympanoplasty | 5 | MHL | 12D | Tymp | Edema | Canal dehiscent | N | Decompression | 1 |
| 37 | Male | 37 | Right | Our | CSOM | Tympanoplasty | 5 | MHL** | 22D | Tymp | Edema | Canal dehiscent | N | Decompression | 2 |
| 38 | Male | 28 | Right | Other | CSOM | CWD | 5 | CHL | 36D | Tymp | Edema | None | N | Decompression | 1 |
| 39 | Male | 7 | Right | Other | CSOM | CWD | 6 | CD | 12D | Tymp, Sg | Completely transected | None | Y | Graft | 3 |
| 40 | Female | 25 | Right | Other | CSOM | CWD | 6 | CD | 8D | Tymp | Completely transected | None | Y | Graft | 3 |
| 41 | Female | 22 | Left | Other | CSOM | Tympanoplasty | 5 | MHL** | 36D | Sg, Mast | Edema | None | N | Decompression | 1 |
| 42 | Female | 35 | Left | Other | CSOM | CWD | 5* | MHL | 6M+ | Tymp, Sg, Mast | Edema | None | N | Decompression | 3 |
| 43 | Female | 52 | Left | Other | CSOM | Tympanoplasty | 6* | CHL** | 3M+ | Sg | Completely transected* | None | N | Graft | 4 |
| 44 | Female | 5 | Right | Our | Congenital atresia of external auditory canal | Canalplasty, Tympanoplasty | 5 | CHL | 2M+ | / | Canal intact, Mild edema | Anomalous course | N | Decompression | 3* |
| 45 | Female | 29 | Right | Other | Congenital atresia of external auditory canal | Canalplasty, Tympanoplasty | 6* | MHL | 6M+ | Sg | Completely transected* | Anomalous course | N | Graft | 5 |

^a^ For 37 patients transferred from other centers, *Primary disease* and *Type of primary surgery* were obtained from medical records.

^b^ Primary disease: *CSOM* Chronic suppurative otitis media, *Chol* Cholesteatoma of middle ear.

^c^ Type of surgery: *CWD* Canal wall down mastoidectomy (including Bondy’s modified radical mastoidectomy), *CWU* Canal wall up mastoidectomy. * Four cases had middle ear surgery history before primary surgery.

^d^ Preoperative grade (H-B): * Four cases had exploration before revision surgery.

^e^ Preoperative hearing: *CHL* conductive hearing loss, *MHL* Mixed hearing loss, *CD* complete deafness. Classified according to 2021 WHO classification of hearing loss. * Reconstructed with partial ossicular replacement prosthesis (PORP), ** reconstructed autologous ossicular chain.

^f^ Duration before surgery (Days or Months): n M+ means between n months to n+1 months.

^g^ Site of facial nerve injury: *Tymp* Tympanic segment, *SG* Second genu, *Mast* Mastoid segment, *GG* Geniculate ganglion.

^h^ Degree of facial nerve injury: Superficially transected (<1/3 of the circumference), partially transected (>1/3 of the circumference). *Developed

traumatic neuroma.

^i^ Final postoperative grade (H-B): * No.44 case suffered from congenital H-B grade II facial nerve paralysis.
